# Fertilization modes and the evolution of sperm characteristics in marine fishes: Paired comparisons of externally and internally fertilizing species

**DOI:** 10.1002/ece3.9562

**Published:** 2022-12-04

**Authors:** Takeshi Ito, Masaya Morita, Seiya Okuno, Kazuo Inaba, Kogiku Shiba, Hiroyuki Munehara, Yasunori Koya, Mitsuo Homma, Satoshi Awata

**Affiliations:** ^1^ Department of Biology, Graduate School of Science Osaka Metropolitan University Osaka Japan; ^2^ Department of Biology and Geosciences, Graduate School of Science Osaka City University Osaka Japan; ^3^ Sesoko Station, Tropical Biosphere Research Center University of the Ryukyus Motobu Japan; ^4^ Shimoda Marine Research Center University of Tsukuba Shimoda Japan; ^5^ Usujiri Fisheries Station, Field Science Center for Northern Biosphere Hokkaido University Hakodate Japan; ^6^ Department of Biology, Faculty of Education Gifu University Gifu Japan; ^7^ Diving Service F. WAVE Sado Japan

**Keywords:** copulation, external fertilization, genital morphology, internal fertilization, marine fish, sperm competition

## Abstract

Fertilization mode may affect sperm characteristics, such as morphology, velocity, and motility. However, there is little information on how fertilization mode affects sperm evolution because several factors (e.g., sperm competition) are intricately intertwined when phylogenetically distant species are compared. Here, we investigated sperm characteristics by comparing seven externally and four internally fertilizing marine fishes from three different groups containing close relatives, considering sperm competition levels. The sperm head was significantly slenderer in internal fertilizers than in external fertilizers, suggesting that a slender head is advantageous for swimming in viscous ovarian fluid or in narrow spaces of the ovary. In addition, sperm motility differed between external and internal fertilizers; sperm of external fertilizers were only motile in seawater, whereas sperm of internal fertilizers were only motile in an isotonic solution. These results suggest that sperm motility was adapted according to fertilization mode. By contrast, total sperm length and sperm velocity were not associated with fertilization mode, perhaps because of the different levels of sperm competition. Relative testis mass (an index of sperm competition level) was positively correlated with sperm velocity and negatively correlated with the ratio of sperm head length to total sperm length. These findings suggest that species with higher levels of sperm competition have faster sperm with longer flagella relative to the head length. These results contradict the previous assumption that the evolution of internal fertilization increases the total sperm length. In addition, copulatory behavior with internal insemination may involve a large genital morphology, but this is not essential in fish, suggesting the existence of various sperm transfer methods. Although the power of our analyses is not strong because of the limited number of species, we propose a new scenario of sperm evolution in which internal fertilization would increase sperm head length, but not total sperm length, and change sperm motility.

## Graphical abstract

We compared sperm characteristics between seven externally and four internally fertilizing species to investigate the evolutionary effect of fertilization mode, considering sperm competition levels. We found that internal fertilization should increase sperm head length, but not total sperm length, and change sperm motility, whereas sperm competition should affect sperm length and velocity. Our findings provide a new perspective on the evolutionary biology of sperm in fishes.

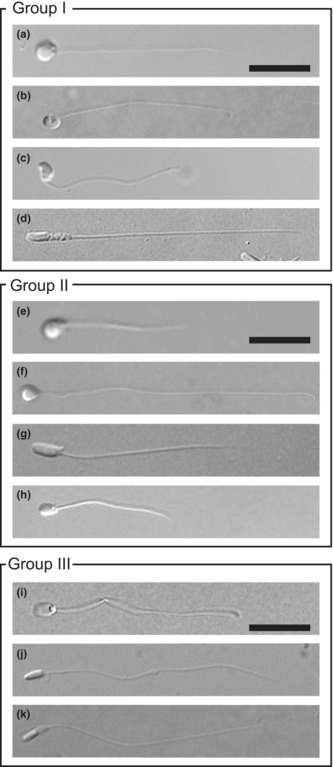

## 1 INTRODUCTION

Spermatozoa exhibit a high degree of variation among animal species with respect to morphology and velocity (Pitnick et al., 2009). Several factors have been suggested to contribute to this variation, such as fertilization mode (i.e., external or internal fertilization; Jamieson, 1991; Mohri et al., 2006), phylogeny (Jamieson, 1987), and postmating sexual selection, including sperm competition (Parker, 1970), and cryptic female choice (Pitnick et al., 2009). Fertilization modes and sperm competition are thought to be evolutionary forces that generate sperm diversity (Immler et al., 2007; Jamieson, 1991; Zeng et al., 2014); however, these factors are confounded when phylogenetically distant species are compared, and the relationship between them and sperm characteristics is obscure in many cases (Lüpold & Pitnick, 2018; Pitnick et al., 2009; Simpson et al., 2014).

Generally, the sperm of internally fertilizing species is believed to be longer than that of externally fertilizing species (Franzén, 1970; Lüpold & Pitnick, 2018; Stockley et al., 1996). A recent study also demonstrated that the fertilization mode may drive sperm length evolution with controlled phylogenetic relationships, both across the animal kingdom and at the phylum and class level (Kahrl et al., 2021). This study discusses differences in sperm size with respect to the raffle and displacement mechanisms of sperm competition related to different body sizes. In addition to explaining these mechanisms by body size, considering the effect of actual sperm competition levels on sperm will provide further insight into sperm evolution through the evolution of fertilization mode. The comparison of larger time scales may also miss local evolutionary directions and may involve various confounding factors, even if phylogeny is taken into account. To consider factors, such as fertilization mode and sperm competition level, it is advisable to compare them in closely related species (Cox & Logan, 2021). However, few taxa contain both externally and internally fertilizing species in closely related species, and the evidence that fertilization modes affect sperm characteristics is still limited by not taking into account fertilization mode and sperm competition levels at the same time (Birkhead et al., 2009; Lüpold & Pitnick, 2018).

Sperm competition is also one of the factors that lead to sperm evolution. Over the past 50 years, many studies on sperm competition have documented that sperm competition affects sperm morphology and velocity, both within and across species (Lüpold et al., 2020; Pitnick et al., 2009; Pizzari & Parker, 2009; Simmons & Wedell, 2020). Comparative studies across taxa have shown that species with a higher risk of sperm competition produce longer and faster‐swimming sperm (e.g., fishes: Fitzpatrick et al., 2009; mammals: Tourmente et al., 2011; birds: Briskie & Montgomerie, 1992; Immler et al., 2011; Lüpold et al., 2009). By contrast, a number of studies have also shown that sperm competition levels are negatively correlated or uncorrelated with sperm length and velocity (Gage et al., 2002; Gage & Freckleton, 2003; Langen et al., 2019; Stockley et al., 1997). These exceptions are taxon‐specific, suggesting that other factors may be relevant. To understand the relationship between sperm evolution and different fertilization modes, it is necessary to consider sperm competition levels, which have a significant effect on sperm traits.

For examining sperm evolution in relation to fertilization modes, fish can be a good model because they exhibit different fertilization modes and various levels of sperm competition, although cartilaginous fish are not suitable because they all perform internal fertilization (Heinicke et al., 2009; Long et al., 2009). Although most teleost species exhibit external fertilization, a few taxa (3%–5% of teleost species) perform internal fertilization with copulatory behavior (Benun Sutton & Wilson, 2019; Fitzpatrick, 2020), which may have evolved multiple times (Goodwin et al., 2002; Reynolds et al., 2002). In addition to normal internal fertilization, several teleosts possess an unusual fertilization mode known as an internal gametic association (IGA) with copulation (Munehara et al., 1989). Only a few teleost fish families exhibit IGA (Evans & Meisner, 2009), including some Aulorhynchidae (Akagawa et al., 2008; Okiyama et al., 1993), Characidae (Pecio et al., 2005), Cottidae (Munehara et al., 1989), and Siluriformes (Parreira et al., 2009). The process by which sperm reaches the egg during IGA is similar to that of internal fertilization (see Section 2). Although species with internal fertilization and IGA are not the majority of teleost fishes, it is possible to compare different fertilization modes (i.e., external vs. internal/IGA) in closely related species.

Previous studies indicated that teleosts with internal fertilization tend to possess elongated sperm heads (i.e., slender heads) and longer sperm than species with external fertilization (Jamieson, 1991; Stockley et al., 1996). However, these studies focused only on the fertilization mode, and sperm competition level was not considered. Similar to internal fertilizing species, the sperm head of IGA species is longer than that of species with external fertilization in marine Cottidae (Baccetti, 1986; Koya et al., 2011; Petersen et al., 2005). However, contrary to the results of these studies, several internally fertilizing fish have oval‐headed sperm (Grier et al., 1978; Ito et al., 2021; Yao et al., 1995), and several externally fertilizing fish have sperm with a slender head (Biagi et al., 2016; Hara & Okiyama, 1998; Mattei, 1991). Therefore, the evolutionary effect of the fertilization mode on sperm characteristics remains unclear, even in teleost fish.

Furthermore, teleosts have various mating systems and a wide range of sperm competition levels (Stockley et al., 1997). As the level of sperm competition increases, total sperm length and velocity increase in Tanganyikan cichlid fish (Balshine et al., 2001; Fitzpatrick et al., 2009). A recent meta‐analysis indicated that the multiple paternity rates of internal fertilizers are higher than those of external fertilizers, suggesting that internal fertilizers can also possess high levels of sperm competition (Fitzpatrick, 2020). Therefore, correctly estimating the level of sperm competition allows for the comparison of sperm from external and internal fertilizing species.

Phylogenetic comparative studies within a family or genus are appropriate for considering phylogenetic effects (John & Nicholas, 1997; Paradis, 2014). However, in addition to the small number of internal fertilizers in teleosts, there is sometimes a lack of information on internal fertilizers and their closely related external fertilizing species for which sperm competition levels can be estimated. Hence, it may be difficult to perform large‐scale phylogenetic comparisons of fertilization modes considering the level of sperm competition. When a sufficient number of samples cannot be obtained because of ecological factors, paired comparisons may be of use (Balshine et al., 2001). In this case, comparing phylogenetically close species and considering overarching information (e.g., ecology and behavior) could effectively infer specific agents of selection that drive adaptation, and complement and even improve on large‐scale phylogenetic comparisons (Cox & Logan, 2021).

**FIGURE 1:**
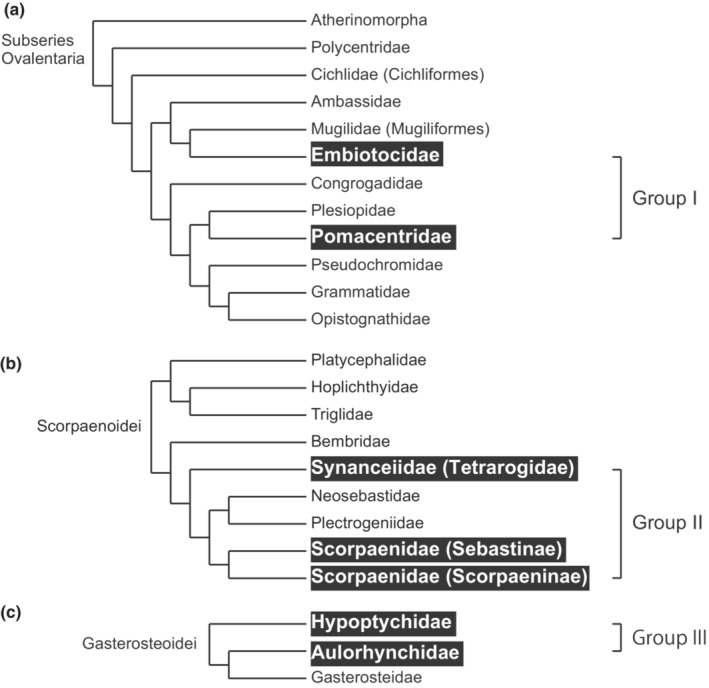
Interfamilial relationship of phylogeny of (a) Ovalentaria, (b) Scorpaenoidei, and (c) Gasterosteoidei. Each phylogenetic tree was based on Near et al. (2013), Smith et al. (2018) and Kawahara et al. (2009), respectively. Outlined taxa show the families used in this study.

Here, we investigated the effect of fertilization mode on sperm characteristics in marine fishes, taking into account the different mating systems and sperm competition levels. We chose three groups of marine fishes containing external and internal fertilizing species in close relatives, for which the levels of sperm competition can be inferred from the reproductive system (Group I: Pomacentridae vs. Embiotocidae, Group II: Synanceiidae vs. Sebastinae vs. Scorpaeninae, and Group III: Aulorhynchidae vs. Hypoptychidae; Figure 1). We analyzed the relationship between the fertilization mode or sperm competition, and sperm characteristics (morphology, velocity, and motility in different solutions). We also examined male genital morphology to determine its relationship with fertilization mode, as the genitalia are typically projected in internally fertilizing species (e.g., mammals, cartilaginous fish, and reptiles). These characteristics were analyzed in paired comparisons within groups of closely related species to control for phylogenetic effects and in phylogenetic comparative methods.

## 2 MATERIALS AND METHODS

### 2.1 Fish sampling and measurements of genital length and testes mass

Focusing on species that can be collected in the seas around Japan, we chose seven species of externally fertilizing fish, three internally fertilizing species, and one IGA species, for which the level of sperm competition could be estimated. They are closely related to each other within the group (see below). In IGA species, males transfer sperm into the ovaries of females by copulation, and sperm swim in the ovarian fluid and come into contact with eggs (Koya et al., 2002; Munehara et al., 1989). Sperm‐egg fusion occurs after the eggs are released into seawater. The fertilization environment is external; however, the process leading up to fertilization is similar to that of internal fertilization. We considered that the selection pressure of IGA on sperm characteristics is almost the same as that of internal fertilization because sperm is transferred to the ovary in the same manner and should adapt to the internal environment. Therefore, we treated the IGA species as an internal fertilizer for the analysis. These 11 species were assigned to three closely related groups (Groups I, II, and III), based on their phylogenetic relationships (Table 1, Figure 1; Kawahara et al., 2009; Near et al., 2013; Nelson et al., 2016; Smith et al., 2018). Group I consisted of externally fertilizing anemonefish (*Amphiprion clarkii*) and damselfish (*Chromis notata* and *Pomacentrus nagasakiensis*) and internally fertilizing surfperch (*Ditrema temmincki temmincki*). Group II comprised externally fertilizing lionfish (*Dendrochirus zebra*) and waspfish (*Paracentropogon rubripinnis*) and internally fertilizing rockfish (*Sebastes cheni* and *Sebastiscus marmoratus*). Group III consisted of externally fertilizing tube snouts (*Aulorhynchus flavidus*), sand eels (*Hypoptychus dybowskii*), and Japanese tube snouts (*Aulichthys japonicus*), which show IGA (Akagawa et al., 2008; Okiyama et al., 1993). We compared the sperm of different fertilization modes within groups of closely related species, but several pairs were compared between families because there are few families in which both fertilization modes are represented (Table 1).

**TABLE 1:** Family and species names of fish, their fertilization mode, mating system, estimation of sperm competition level (ESCL), and relative testes mass (RTM)

Group	Family (subfamily)	Species	Fertilization mode	Mating system	Sneaker	Density of mating site	Frequency of multiple mating	ESCL	RTM (number of individuals)	References
I	Pomacentridae	*Amphiprion clarkii*	External	Monogamy	Absent	Low	Low	Low	−0.49 ± 0.05 (7)	Moyer and Bell (1976), Moyer and Nakazono (1978) and Ochi (1989)
		*Chromis notata*	External	MTV polygamy	Present?	High	Low	Medium	0.29 ± 0.28 (20)	Ochi (1985, 1986) and Picciulin et al. (2004)
		*Pomacentrus nagasakiensis*	External	MTV polygamy	Present?	High	Low	Medium	0.37 ± 0.1 (8)	Moyer (1975)
	Embiotocidae	*Ditrema temmincki temmincki*	Internal	MTV polygamy	Present?	High	High	High	−0.07 ± 0.31 (15)	Izumiyama et al. (2020), Nakazono and January (1981) and Takagi et al. (2008)
II	Scorpaenidae (Scorpaeninae)	*Dendrochirus zebra*	External	Promiscuity^a^	Absent	Low	Low	Low	−0.5 ± 0.12 (10)	Moyer (1981)
	Synanceiidae (Tetrarogidae)	*Paracentropogon rubripinnis*	External	Promiscuity	Present	High	High	High	0.49 ± 0.13 (10)	Matsuoka (2005)
	Scorpaenidae (Sebastinae)	*Sebastes cheni*	Internal	MTV polygamy?^b^	Present	Medium	Medium?	Medium	−0.29 ± 0.36 (7)	Blanco Gonzalez et al. (2009), Coleman and Jones (2011), Shinomiya and Ezaki (1991) and Sogard et al. (2008)
		*Sebastiscus marmoratus*	Internal	MTV polygamy (Promiscuity)	Absent	Low	Low	Low	−0.36 ± 0.13 (5)	Fujita and Kohda (1996) and Ng et al. (2003)
III	Aulorhynchidae	*Aulorhynchus flavidus*	External	MTV polygamy	Absent^c^	High	No data	Medium	0.23 ± 0.15 (4)	Limbaugh (1962) and Schram and Allen (2014)
	Hypoptychidae	*Hypoptychus dybowskii*	External	MTV polygamy	Present	High	High	High	0.19 ± 0.08 (6)	Akagawa and Okiyama (1993) and Narimatsu and Munehara (2001)
	Aulorhynchidae	*Aulichthys japonicus*	IGA	MTV polygamy	Absent	High	Medium	Medium	0.06 ± 0.11	Akagawa et al. (2008, 2004) and Okiyama et al. (1993)

Abbreviation: IGA, Internal gametic association; MTV polygamy, Male‐territory‐visiting polygamy.

^a^ The mating system of *D. zebra* has been reported to be promiscuous, but mating occurs between dyadic relationships (i.e., monogamous). Therefore, the level of sperm competition was predicted to be low.

^b^ Closely related species *S. inermis* and *S. atrovirens* showed male‐territory‐visiting polygamy and multiple paternities.

^c^ Aquarium observations revealed the occurrence of sneaker males (Takamizo et al., 2015), but we referred to field studies to arrange the conditions among species, as other studies were conducted in the field.

As data from disparate sources may differ in quality and may be erroneous (Freckleton, 2009), we caught all fish in the field, except for *A. flavidus*, and used them for the following analyses. Fish were collected by hand nets using SCUBA, hook and line, and gillnets during the reproductive season in the nearshore waters of Japan and off the west coast of California, USA (Table S1). Three *A. flavidus* were obtained from an aquarium in Tokyo Sea Life Park, Japan because we could not access the natural habitat, off the Pacific coast of North America, owing to the COVID‐19 pandemic. Note that they were bred in the tank, but the tank was close to natural conditions. The specimens were anesthetized using MS‐222 (200 mg/L) and euthanized through cervical transection by snipping the spinal cord between cervical vertebrae 1 and 2 with sharp scissors, according to the American Veterinary Medical Association. Total length (mm), standard length (mm), and body mass (g) were measured. We photographed the genital papilla by gently pressing the abdomen to observe male genital morphology. The length of the genital papilla (mm) from the basal position to the tip was measured using photographs and Image J ver. 1.50 (National Institutes of Health). Testes or ovaries of the specimens were removed and weighed (g).

### 2.2 Sperm motility and velocity

The extracted testes were placed on ice in a Petri dish to prevent contamination with urine, mucus, and seawater. Sperm were collected by incising the posterior region of the testes, which contained mature sperm prior to ejaculation, and were used for subsequent analysis. We immediately diluted the extracted semen (<1 μl) in natural seawater or isotonic solution (30 μl) to simulate the osmotic pressure of ovarian fluid (150 mM NaCl and 10 mM HEPES, pH 8.0; Ito & Awata, 2019; Koya et al., 1993) on glass slides coated with 1% bovine serum albumin. Although it is not known if the isotonic solution was the best solution for all species, at least sperm were motile normally (i.e., neither unnatural flagellar movements nor apparently strange head vibrations were observed). Sperm motility was observed in both seawater and isotonic solution using a phase‐contrast microscope (DSM‐IIH‐104, Daiko Science) equipped with a digital CCD camera (MTV‐63WIN, Mintron Enterprise), and the sperm motility was recorded using the Blackmagic Video Recorder software ver. 1.0.1 (Blackmagic Design) and AmScope ver. 4.8 (AmScope). One female out of 10 individuals in internally fertilizing *D. temmincki temmincki* and one female out of seven individuals in *A. japonicus* were used for sperm measurements (Table S2) using the same method as above because ovarian fluid contained sperm. Although the effect of ovarian fluid on sperm has not been studied in these two fish species, all sperm swimming speed from the ovary were within the range of that from the testes (see Tables [Link], [Link], and S6).

We measured the sperm velocity of externally fertilizing species in seawater and internally fertilizing species in an isotonic solution from recorded videos. Water temperature was set according to the seawater temperature at which the fish were collected (Table S1). Sperm trajectories were recorded for 1 s (30 frames/s) using cell motility analysis software (BohBoh ver. 4.51 J, BohBoh Soft) during 0–30 s of the videos, and sperm curvilinear velocities were measured using sperm trajectories and Image J ver. 1.50i.

### 2.3 Sperm morphology

Sperm collected from the posterior region of the testes or ovarian fluid was fixed with optimal fixative (2.5% glutaraldehyde, 0.45 M glucose, 60 mM HEPES; Ito & Awata, 2019). The sample was placed on a microscope slide, and images of sperm morphology were obtained using a differential interference microscope (BX50, Olympus) equipped with a digital color CCD camera (DMK 33UX174, The Imaging Source) and IC capture software ver. 2.4 (The Imaging Source), or a differential interference microscope (BX53, Olympus), equipped with a digital color CCD camera (DP73, Olympus) and CellSens Standard software ver. 1.9 (Olympus). We measured the morphology of the sperm components (total sperm length, flagellum length, head length, head width, midpiece length, midpiece width, head length/width, midpiece length/width, and head length/total sperm length) in the images using ImageJ ver. 1.50i. The head length/width and midpiece length/width ratios were used to determine head and midpiece morphology.

### 2.4 Estimation of sperm competition levels

To increase the resolution of this study, two approaches were applied to estimate sperm competition levels. First, the degree of sperm competition was estimated based on the situational and ecological contexts in which sperm competition can actually occur. For example, different mating systems generate different levels of sperm competition (Dunn et al., 2001; Harcourt et al., 1995; Smith, 1984): low levels of sperm competition in monogamous species that present a dyadic relationship and high levels in promiscuous or polyandrous species that mate with multiple partners (Birkhead, 1998; Taborsky, 1998). The presence of sneaker males also increases the level of sperm competition (Kustra & Alonzo, 2020; Montgomerie & Fitzpatrick, 2009). In addition, high population densities of fish increase sperm competition levels (Liu et al., 2013; Soucy & Travis, 2003). We compiled ecological information on multiple aspects to estimate the level of sperm competition and categorized sperm competition into three levels: low, medium, and high (Table 1).

Second, we estimated sperm competition levels from the relative testis mass (RTM). Although RTM inherently reflects the outcome of sperm competition and does not infer sperm competition levels, it is generally used as a proxy for sperm competition. Species with higher levels of sperm competition are predicted to have larger testes than those with lower levels of sperm competition (e.g., Gage & Freckleton, 2003; Rowley, Daly‐Engel, et al., 2019; Rowley, Locatello, et al., 2019; Simmons & Fitzpatrick, 2016; Vicens et al., 2014; Zeng et al., 2014). In addition, RTM is associated with differences in mating systems (Baker et al., 2019). We calculated the RTM from the regression line of body mass and testes mass (see Section 2.6) and treated RTM as the risk of sperm competition.

### 2.5 Construction of phylogenetic trees

We conducted paired comparisons of the sperm characteristics within the three groups and performed evolutionary analyses using phylogenetic comparative methods. To conduct evolutionary analyses of sperm characteristics according to fertilization mode and sperm competition, we extracted 11 species from phylogenetic trees of two previous studies (Betancur et al., 2017; Rabosky et al., 2013) and generated two phylogenetic trees using the R packages “ape” (Paradis et al., 2004) and “phytools” (Revell, 2012). Four species (*Chromis notata*, *Pomacentrus nagasakiensis*, *Paracentropogon rubripinnis*, and *Sebastes cheni*) were not included in the trees; therefore, different species of the same genus were used instead. Specifically, *Paracentropogon* did not occur in either tree. Instead of *P. rubripinnis*, we used *Ablabys taenianotus* in the phylogenetic tree of Rabosky et al. (2013) and *Coccotropsis gymnoderma* in that of Betancur et al. (2017), both of which are members of the family Tetrarogidae. Therefore, the phylogenetic relationships in these trees were expected to be similar to those in trees based on the original species because each substitute species represents the same family or genus as the original species. The trimmed tree described by Rabosky et al. (2013) contained all 11 species. However, *P. rubripinnis* (*A. taenianotus*, Group II) was assigned as a sister taxon to the Gasterosteiformes (Group III). By contrast, the trimmed tree of Betancur et al. (2017) showed that all groups were monophyletic, and the taxonomy was maintained. However, this tree does not include the genus *Sebastiscus*. Therefore, we merged the divergence time between *S. marmoratus* and *Sebastes cheni* of the trimmed tree of Rabosky et al. (2013) with that of Betancur et al. (2017) and used it for further analyses (the merged tree is shown in Figure S1).

### 2.6 Data analyses

Although we collected mature sperm from the posterior region of the testis, to rule out the possibility of measuring immature sperm, we eliminated outliers from the datasets using the IBM SPSS Statistics ver. 23.0 (SPSS, Inc.) before analyses (50 out of 1262 [3.9%] sperm for morphology and 40 out of 1689 sperm [2.4%] for velocity, Tables [Link], [Link]). We compared sperm characteristics, including total sperm length, head length and width, midpiece length and width, and velocity among external and internal fertilizers within the same group (Groups I, II, or III). Analyses were conducted on three groups using linear mixed models (LMMs) as implemented in the “lme4” package (Bates et al., 2015) with R 3.4.1 (R Core Team, 2016). In the models, we set “species” as a fixed effect and “individual ID” as a random effect because we measured sperm characteristics of multiple sperm per individual. Multiple comparisons among species were conducted using Tukey's all‐pair comparisons with the “multcomp” package (Hothorn et al., 2016) to control family‐wise error rates among species. Differences were considered statistically significant at *p* < .05.

In addition to the LMM analyses, we also performed a phylogenetic generalized least squares (PGLS) approach (Freckleton et al., 2002; Pagel, 1999) to improve the resolution of the research and to reveal the relationship between fertilization mode or sperm competition and sperm characteristics in a phylogenetic context. Although our sample size was small, a model with a few predictors (two or three) might still reveal reasonable results with a considerably small dataset (Mundry, 2014), and our models focused on two predictors. We used fertilization mode and sperm competition levels as independent variables in each model. An interaction term between independent variables was not incorporated into the model, as our dataset was not sufficiently large. PGLS analyses were conducted using the R package “caper” (Orme et al., 2013) in a maximum‐likelihood framework. To assess the phylogenetic nonindependence of the averaged species data, Pagel's lambda was estimated according to the ML estimate (0: low phylogenetic signal; 1: high phylogenetic signal), although the lambda estimates were less robust when the sample size was <20 species (Freckleton et al., 2002).

For the index of sperm competition, we calculated the residuals of all individuals as RTM from the regression line estimated by the LMM based on the relationship between log_10_ soma mass (body mass—visceral mass—testes mass) and log_10_ testes mass, setting species as a random effect (Figure S2). We performed a PGLS regression analysis between RTM and sperm characteristics to assess whether the species in which males invested more in the testes would have superior sperm (i.e., faster‐swimming sperm). We also examined the relationship between sperm velocity and morphological components using the PGLS approach.

To assess whether males of internally fertilizing species had elongated genitalia, we calculated the relative genital length. The relative genital length was estimated as the residual from the regression line estimated by an LMM on the relationship between log_10_ standard length (mm) and log_10_ genital length (mm), and the species ID was set as a random effect (Figure S3). The mean relative genital length was compared using ANOVA, followed by the Tukey's HSD test for multiple comparisons. We also examined whether relative genital length was related to fertilization mode and sperm competition level using the PGLS approach.

## 3 RESULTS

### 3.1 Sperm morphology

**FIGURE 2:**
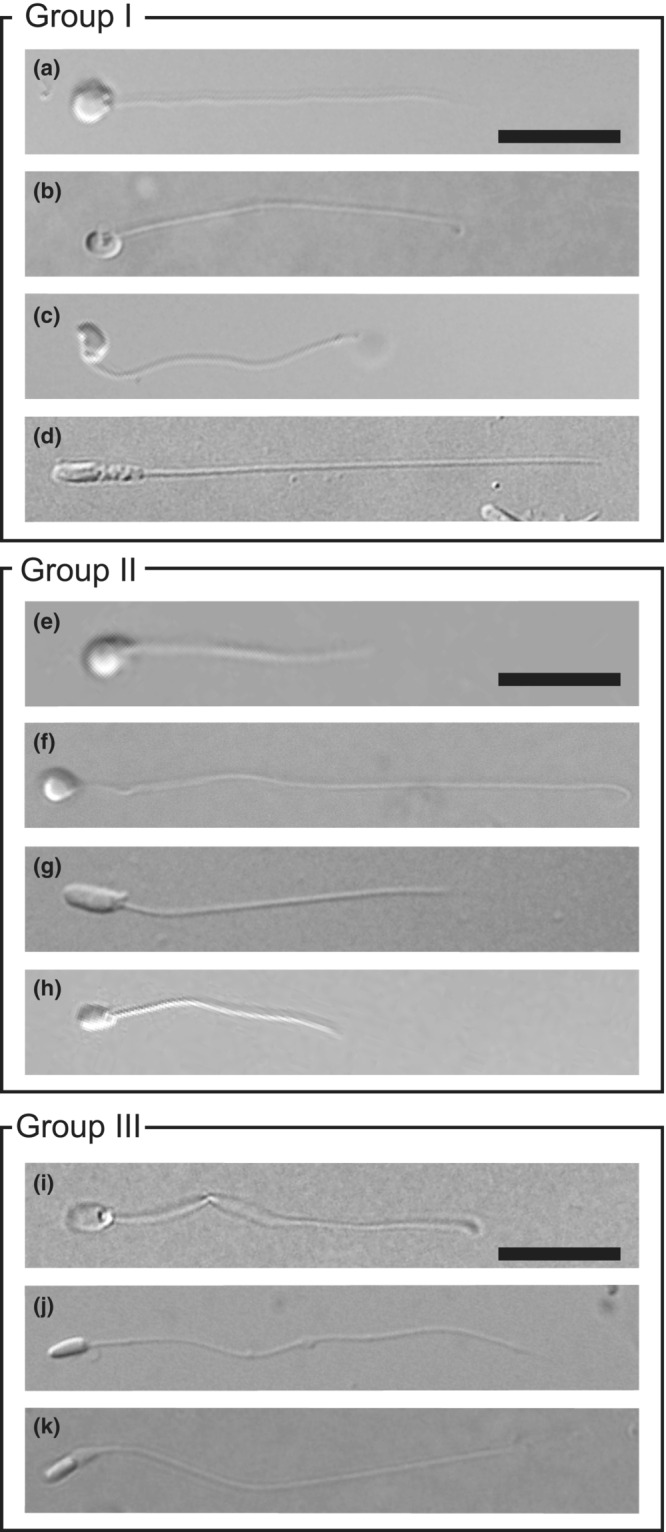
Sperm morphology of species with external and species with internal insemination. (a) *Amphiprion clarkii*. (b) *Chromis notata*. (c) *Pomacentrus nagasakiensis*. (d) *Ditrema temmincki temmincki*. (e) *Dendrochirus zebra*. (f) *Paracentropogon rubripinnis*. (g) *Sebastes cheni*. (h) *Sebastiscus marmoratus*. (i) *Aulorhynchus flavidus*. (j) *Hypoptychus dybowskii*. (k) *Aulichthys japonicus*. Scale bars indicate 5 mm.

**FIGURE 3:**
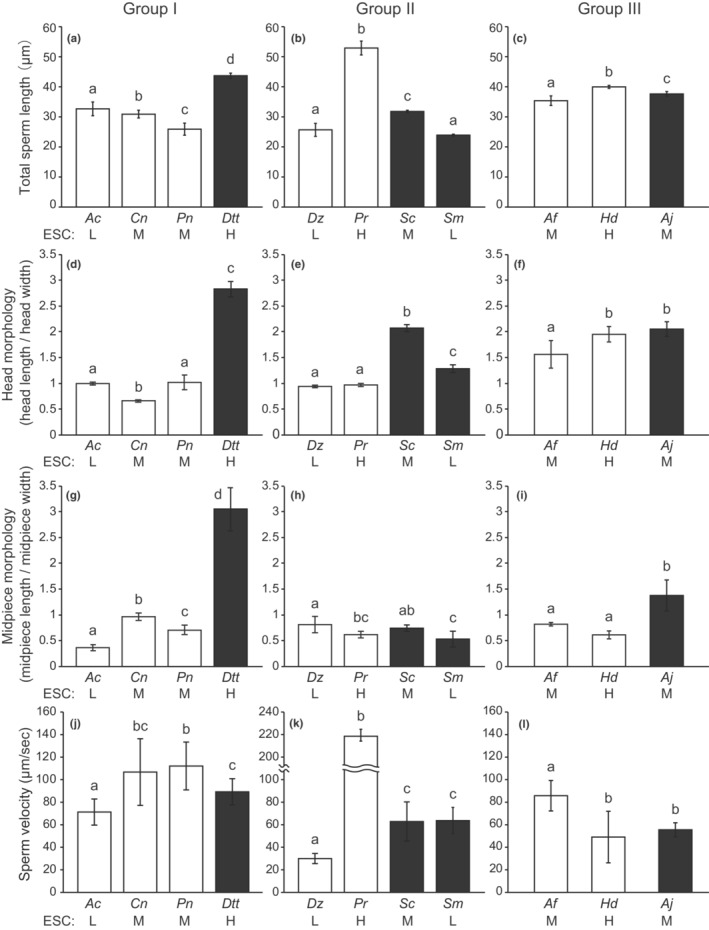
Differences in sperm morphological characteristics and sperm velocity (mean ± SD) of three groups with different fertilization modes. Total sperm length in Group I (a), Group II (b), and Group III (c). Head ratio in Group I (d), Group II (e), and Group III (f). Midpiece ratio in Group I (g), Group II (h), and Group III (i). Sperm velocity in Group I (j), Group II (k), and Group III (l). White bars and black bars indicate species with external fertilization and internal insemination, respectively. Estimated relative sperm competition levels (ESCL) are also shown under the bar: Low (L), medium (M), and high (H). *Ac*, *Amphiprion clarkii*; *Af*, *Aulorhynchus flavidus*; *Aj*, *Aulichthys japonicus*; *Cn*, *Chromis notata*; *Dtt*, *Ditrema temmincki temmincki*; *Dz*, *Dendrochirus zebra*; *Hd*, *Hypoptychus dybowskii*; *Pn*, *Pomacentrus nagasakiensis*; *Pr*, *Paracentropogon rubripinnis*; *Sc*, *Sebastes cheni*; *Sm*, *Sebastiscus marmoratus*. Different letters indicate significant differences between species (LMMs with sequential Bonferroni correction, *p* < .05).

Sperm morphology was diverse among species with external and internal fertilization, including IGA (Figure 2). In Group I, the total sperm and flagella lengths of internally fertilizing species with higher estimated sperm competition levels were significantly longer than those of externally fertilizing species (Figure 3A, Table S4). However, there was no apparent relationship between fertilization mode and total sperm length or flagella length in Groups II and III. Significant differences in total sperm length and flagella length were detected in Groups II (Figure 3B, Table S5) and III (Figure 3C, Table S6), and these traits seemed to be related to sperm competition. PGLS analyses also showed that the total sperm length and flagella length increased evolutionarily with increasing levels of sperm competition (Table 2).

**TABLE 2:** PGLSs of fertilization mode (FM) and estimated sperm competition levels (ESCL) affecting sperm characteristics

Traits	*λ*	95% CI	Adjusted *R* ^2^	Predictors	*F*	*p*
Total sperm length (μm)	0^1, 0.01^	NA–0.67	.60	FM	0.02	.89
				**ESCL**	**8.87**	**.01**
Flagella length (μm)	0^1, 0.01^	NA–0.70	.59	FM	0.13	.73
				**ESCL**	**8.74**	**.01**
Head length (μm)	0^1, 0.004^	NA–0.60	.13	FM	4.05	.08
				ESCL	0.22	.81
Head width (μm)	1^0.03, 1^	0.33–NA	.49	**FM**	**6.27**	**.04**
				ESCL	3.07	.11
Midpiece length (μm)	1^0.09, 1^	NA–NA	.22	FM	5.29	.06
				ESCL	0.29	.75
Midpiece width (μm)	0^1, 0.003^	NA–0.65	.15	FM	0.06	.82
				ESCL	2.33	.17
Head length/head width	0^1, 0.14^	NA–NA	.59	**FM**	**11.36**	**.01**
				ESCL	2.97	.11
Midpiece length/midpiece width	1^0.09, 1^	NA–NA	.18	FM	3.90	.09
				ESCL	0.63	.56
Sperm velocity (μm/s)	0^1, 0.30^	NA–NA	.03	FM	0.83	.39
				ESCL	1.25	.34

*Note*: Superscripts following the *λ* estimates represent the significance levels of the likelihood‐ratio tests (first position: against *λ* = 0; second position: against *λ* = 1). The adjusted *R* ^2^ and 95% confidence intervals (CIs) were calculated for each model. Bold prints indicate statistically significant correlations.

Sperm head morphology differed between externally and internally fertilizing/IGA species in all the groups (Figure 2). Sperm heads of internal fertilizers/IGA species were more slender than those of external fertilizers, which showed spherical or oval morphology (Figure 3D–F; Tables [Link], [Link]). One exception was *Hypoptychus dybowskii* in Group III (Figure 3F, Table S6), which had a slightly longer sperm head than the other two externally fertilizing species. However, no statistical difference in the head ratio was found between *A. japonicus* and *H. dybowskii*. PGLS analysis showed that head morphology was elongated by the evolution of fertilization modes from external to internal, and was not related to sperm competition (Table 2).

The ratio of midpiece length to width showed similar results to head morphology, in which internally fertilizing/IGA species had a longer midpiece than externally fertilizing species in groups I and III (Figure 3G,I, Tables S4 and S6). Nevertheless, there was no trend between midpiece morphology and fertilization mode or sperm competition in Group II (Figure 3H, Table S5). The PGLS analysis also showed a (nonsignificant) trend of a positive evolutionary relationship between the midpiece ratio and fertilization mode (Table 2).

### 3.2 Sperm motility and velocity

Sperm motilities in different solutions differed between external and internal fertilizers/IGA in all three groups (Table 3). The sperm of externally fertilizing species were motile in seawater but immotile in an isotonic solution that imitated ovarian fluid. By contrast, sperm of internally fertilizing species, including IGA species, were motile in isotonic solution but immotile in seawater. Only the sperm of *Aulorhynchus flavidus* was active in both seawater and isotonic solutions (Table 3).

**TABLE 3:** Sperm motility in seawater and isotonic solution

Group	Species	Fertilization mode	Sperm motility	
			Seawater	Isotonic solution
I	*Amphiprion clarkii*	External	Motile	Immotile
	*Chromis notata*	External	Motile	Immotile
	*Pomacentrus nagasakiensis*	External	Motile	Immotile
	*Ditrema temmincki temmincki*	Internal	Immotile	Motile
II	*Dendrochirus zebra*	External	Motile	Immotile
	*Paracentropogon rubripinnis*	External	Motile	Immotile
	*Sebastiscus cheni*	Internal	Immotile	Motile
	*Sebastes marmoratus*	Internal	Immotile	Motile
III	*Aulorhynchus flavidus*	External	Motile	Motile
	*Hypoptychus dybowskii*	External	Motile	Immotile
	*Aulichthys japonicus*	IGA	Immotile	Motile

Abbreviation: IGA, Internal gametic association.

We did not obtain consistent results between sperm velocity and fertilization mode (Figure 3; Tables [Link], [Link]). Regarding sperm competition, in Group I, the sperm velocity of *A. clarkii*, in which the sperm competition level was low, was lower than that of other species (Figure 3J, Table S4). In Group II, *P. rubripinnis*, facing higher levels of sperm competition, produced markedly faster sperm than the other species with low or medium levels of sperm competition (Figure 3K, Table S5). However, no trend between sperm velocity and sperm competition level was observed in Group III; *A. flavidus* had faster sperm than the other two species (Figure 3L, Table S6). No relationship was found between sperm velocity and fertilization mode or sperm competition levels using the PGLS approach (Table 2).

### 3.3 Relationships between RTM and sperm characteristics

**FIGURE 4:**
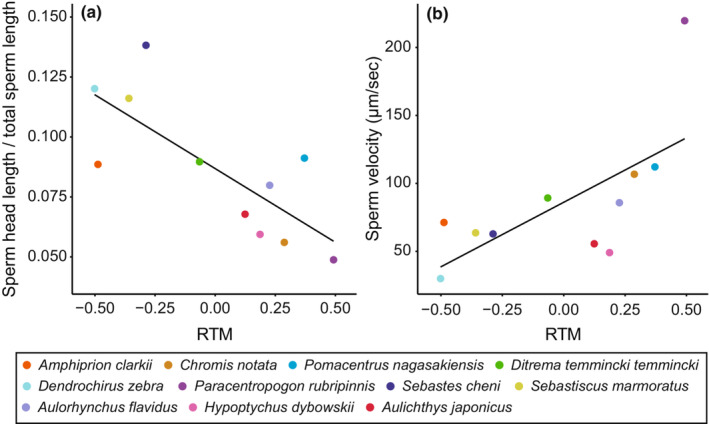
Relationship between relative testes mass (RTM) and sperm components. (a) Correlation between RTM and the ratio of sperm head length to total sperm length (PGLS, intercept = 0.08, slope = −0.061). (b) Correlation between RTM and velocity (PGLS, intercept = 82.49, slope = 92.97). Regression lines are from PGLS analyses (see Table 4 for statistics).

Phylogenetic generalized least squares analysis revealed that the ratio of sperm head length to total sperm length was negatively correlated with RTM (Figure 4A, Table 4), whereas sperm velocity was positively correlated with RTM (Figure 4B). No correlation was detected for the other sperm components, except for the midpiece width (Table 4). We also found that total sperm length was positively correlated with sperm velocity, although this relationship was no longer significant when the outlier (*P. rubripinnis*) was eliminated (PGLS; *F* = 0.02, *p* = .90).

**TABLE 4:** Relationships between sperm characteristics and relative testes mass (RTM) and between sperm velocity and sperm morphological components

Characteristic	Predictor	95% CI	Estimate ± SE	Multiple *R* ^2^	*F*	*λ*	*p*
Total sperm length	RTM	NA–NA	12.40 ± 6.97	.26	3.17	0.0^1, 0.14^	.11
Flagella length	RTM	NA–NA	13.25 ± 6.72	.30	3.89	0.0^1, 0.13^	.08
Head length	RTM	NA–0.91	−0.84 ± 0.51	.23	2.71	0.0^1, 0.02^	.13
Head width	RTM	NA–NA	−0.33 ± 0.39	.07	0.70	1.0^0.06, 1^	.43
Midpiece length	RTM	NA–NA	−0.15 ± 0.53	.008	0.08	1.0^0.11, 1^	.79
Midpiece width	**RTM**	**NA–NA**	−0.82 ± 0.32	**.42**	**6.54**	**0.04** ^**0.92, 0.22**^	**.03**
Head length/head width	RTM	NA–NA	−0.10 ± 0.48	.005	0.05	0.97^0.20, 0.91^	.84
Midpiece length/midpiece width	RTM	NA–NA	0.21 ± 0.48	.02	0.19	1.0^0.05, 1^	.67
Head length/total sperm length	**RTM**	**NA–0.81**	−0.05 ± 0.01	**.59**	**12.97**	**0.0** ^**1, 0.02**^	**.006**
Sperm velocity	**RTM**	**NA–NA**	**82.49** ± **27.46**	**.50**	**9.03**	**1.0** ^**0.28, 1**^	**.02**
	**Total sperm length**	**NA–NA**	**3.61 ± 1.53**	**.38**	**5.55**	**0.0** ^**1, 0.15**^	**.04**
	Head length/Total sperm length	NA–NA	−1142 **±** 592	.29	3.72	0.0^1, 0.37^	.09
	Head length	NA–NA	−16.05 ± 26.55	.04	0.37	0.0^1, 0.75^	.56
	Midpiece length	NA–NA	−4.30 ± 21.64	.004	0.04	0.0^1, 1^	.85

*Note*: Bold print indicates statistically significant correlations.

### 3.4 Genital morphology

**FIGURE 5:**
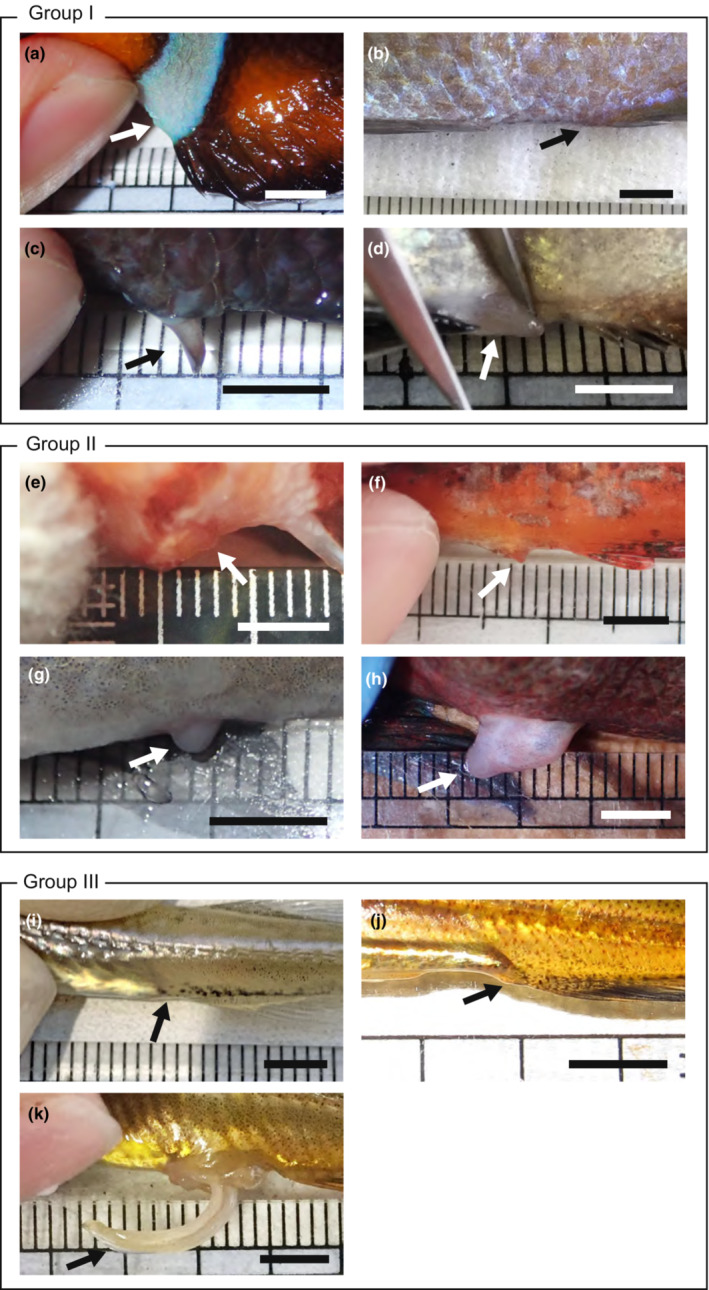
Genital morphology of species with external fertilization and species with internal insemination. (a) *Amphiprion clarkii*. (b) *Chromis notata*. (c) *Pomacentrus nagasakiensis*. (d) *Ditrema temmincki temmincki*. (E) *Dendrochirus zebra*. (f) *Paracentropogon rubripinnis*. (g) *Sebastes cheni*. (h) *Sebastiscus marmoratus*. (i) *Aulorhynchus flavidus*. (j) *Hypoptychus dybowskii*. (k) *Aulichthys japonicus*. Scale bars indicate 5 mm; arrows indicate the positions of genitalia.

As expected, six of the seven external fertilizers did not have apparent genitalia (Figure 5A,B,E,F,I,J, Table 5). Nevertheless, a relatively sizeable genital papilla appeared in *P. nagasakiensis* after pressing the abdomen (Figure 5C). Of the internal fertilizers and species with IGA, *S. marmoratus* and *A. japonicus* had large and slender genitalia that were retracted in the abdominal cavity unless the abdomen was pressed (Figure 5H,K). The other internally fertilizing fishes, such as *D. temmincki temmincki* and *S. cheni*, had no noticeable copulatory organs, although a small genital papilla was present (Figure 5D,G). Although significant differences in relative genital length were detected between species in all three groups (Table 5), the PGLS approach showed that neither fertilization mode (*F* = 2.02, *p* = .20) nor sperm competition level (*F* = 0.31, *p* = .75) affected the relative genital length (*λ* = 0^1, 0.02^, 95% CI = NA–0.764).

**TABLE 5:** Fertilization mode and relative genital length in male fish

Group	Species	Fertilization mode	Relative genital length (*n*)	ANOVA
I	*Amphiprion clarkii*	External	−0.35 ± 0.20^a^ (4)	*F* = 21.80 *p* < .001
	*Chromis notata*	External	−0.15 ± 0.03^ac^ (3)	
	*Pomacentrus nagasakiensis*	External	0.69 ± 0.13^b^ (3)	
	*Ditrema temmincki temmincki*	Internal	0.02 ± 0.21^c^ (5)	
II	*Dendrochirus zebra*	External	−0.64 ± 0.23^a^ (2)	*F* = 43.79 *p* < .001
	*Paracentropogon rubripinnis*	External	0.32 ± 0.10^b^ (8)	
	*Sebastes cheni*	Internal	−0.03 ± 0.13^c^ (6)	
	*Sebastiscus marmoratus*	Internal	0.24 ± 0.09^b^ (9)	
III	*Aulorhynchus flavidus*	External	−1.02 ± 0.11^a^ (3)	*F* = 735.36 *p* < .001
	*Hypoptychus dybowskii*	External	−0.25 ± 0.17^b^ (2)	
	*Aulichthys japonicus*	IGA	1.15 ± 0.02^c^ (6)	

*Note*: Different superscripts indicate significant differences according to the Tukey's HSD test (*p* < .05) among species in each group. The number of individuals is shown in parentheses.

## 4 DISCUSSION

Previous studies on teleost fish have suggested that internal fertilizers, including IGA species, have sperm with a more elongated head than external fertilizers (Jamieson, 1991; Koya et al., 2011). Notably, our results suggest the same tendency as observed in previous studies, even when close relatives were compared, and phylogeny was controlled. Thus, different fertilization patterns could be a strong evolutionary force affecting the sperm head, and we demonstrated the effect of fertilization modes on sperm head morphology. Sperm head morphology affects sperm swimming behavior (Muto & Kubota, 2009; Støstad et al., 2018). This is probably because a narrow head reduces drag so that slender‐headed sperm in internal fertilizing/IGA species can travel easily through the viscous ovarian fluid (Malo et al., 2006; Tourmente et al., 2011; Watanabe et al., 2000). In addition, the viscosity of the ovarian fluid is 2 to 3‐fold higher than that of water (Zadmajid et al., 2019). The theoretical model indicates that the drag of the head ratio around two (i.e., 2:1 head length:head width ratio) is lower than that of the spherical morphology (i.e., 1:1 ratio), although a more slender head (ratio exceeding 4:1) increases the drag more than a sphere (Humphries et al., 2008). In this study, the head ratios of the internal fertilizers were 1.29–2.83 (see Tables [Link], [Link]). The hypothesis that the sperm head of a species undergoing internal insemination is elongated to adapt to a viscous environment is likely applicable to the fish used in the current study. Generally, the ovarian lumen has a somewhat complex structure and is narrow; sperm motility may be more obstructed in the ovary than in seawater, which is an unobstructed space. Therefore, an elongated head may be more suitable for propulsion in structurally complex ovarian environments.

Nevertheless, there was no statistically significant difference between the head morphology of externally fertilizing *H. dybowskii* and *A. japonicus* with IGA in Group III. This result coincides with the observation of sperm morphology in Gasterosteoidei (Hara et al., 2013; Hara & Okiyama, 1998). The selective pressure of the viscous ovarian fluid on sperm may also be expected to act on species with external fertilization because spawned eggs are covered by viscous ovarian fluid (Zadmajid et al., 2019). The elongated head in externally fertilizing *H. dybowskii* may also adapt to the viscous fluid, although externally fertilizing *A. flavidus* with an oval head have a reproductive mode similar to that of *H. dybowskii* (Akagawa & Okiyama, 1993; Limbaugh, 1962). In Baikal sculpins, the sperm of externally and internally fertilizing species also have the same head morphology, whereas the head volume is smaller in internally fertilizing than in externally fertilizing species, which may be an adaptation to viscous environments (Ito et al., 2021). A smaller sperm head has a lower drag force against a solution than a larger head (Marcos et al., 2014). In the present study, there was no difference in the cross‐sectional area of the head between *H. dybowskii* and *A. japonicus* (Table S6). The reason why sperm head morphology was similar between *H. dybowskii* and *A. japonicus* remains unknown, and further detailed research is required to clarify this issue.

Although the sample size was limited in this study, LMM and PGLS analyses support that total sperm length (including flagella length) and velocity varied and may be independent of fertilization mode, in contrast to head morphology in both analyses. Comparative studies among a wide range of taxa have shown that internal fertilizers tend to have longer sperm than external fertilizers (Franzén, 1970; Lüpold & Pitnick, 2018). In bony fish, a previous study in which phylogeny was partially considered indicated a similar result (Stockley et al., 1996). A recent meta‐analysis of a wide range of animals also showed that internal fertilizers have longer sperm than external fertilizers (Kahrl et al., 2021), and there was higher power to detect sperm length differences, although the actual sperm competition levels were not considered. Our comparisons with close relatives and PGLS analysis were weak power owing to sample size but indicated that fertilization mode might not affect sperm length. This result is partially consistent with that of Kahrl et al. (2021); sperm length was not statistically significant in the clade of bony fish, although sperm length was 1.2‐fold larger in internal fertilizer than in external fertilizer. We also showed that the fertilization mode and sperm competition level did not influence sperm velocity, even though their swimming environments were different. Several studies among related species have shown that the total sperm length and velocity increase as sperm competition levels increase (Balshine et al., 2001; Fitzpatrick et al., 2009; Immler et al., 2011; Kleven et al., 2009; Tourmente et al., 2011). Our paired comparison results of total sperm length and velocity were partially consistent with these studies; species with a low level of sperm competition produced shorter sperm than species with high levels of sperm competition (e.g., *A. clarkii* vs. *D. temmincki temmincki* and *D. zebra* vs. *S. cheni*), although some pairs did not confirm this assumption (e.g., sperm length: *A. clarkii* vs. *C. notata* and *P. nagasakiensis*; velocity: *S. cheni* vs. *S. marmoratus*), and there was no relationship between sperm total length and velocity. Overall, the PGLS analysis showed that total sperm length was correlated with sperm competition level. The long flagellum (but not the extra‐long flagellum) is advantageous for swimming speed (Ishijima et al., 1998), such that species with high levels of sperm competition may have longer flagella (Ball & Parker, 1996).

We showed that RTM, a proxy for sperm competition, did not correlate with the total sperm length. However, the ratio of head to total sperm length was negatively correlated with RTM, suggesting that sperm competition promotes relatively longer sperm relative to the head length. The theoretical model predicts that sperm with small heads relative to their total length reduces the drag force to swim in water; thus, relatively longer flagella swim faster (Humphries et al., 2008). The sperm velocity was also positively correlated with RTM. Thus, increasing sperm competition levels may promote a relatively longer flagella‐to‐head length and faster sperm velocity, and the results are likely to match the theory of sperm competition (Ball & Parker, 1996; Parker, 1993).

There was no apparent relationship between the midpiece morphology and fertilization mode or sperm competition. The midpiece contains mitochondria, which generate energy; thus, midpiece volume is likely to be related to sperm velocity (Anderson & Dixson, 2002; Firman & Simmons, 2010; Gil et al., 2009; Tourmente et al., 2009, 2011), and midpiece size is positively correlated with different levels of sperm competition (Anderson et al., 2005; Anderson & Dixson, 2002; Tourmente et al., 2009, 2011). Furthermore, one study reported that different tactics (i.e., sneaker and courting) affect sperm midpiece morphology in internally fertilizing *Xiphophorus nigrensis* (Smith & Ryan, 2010). However, our results showed that midpiece size was not related to the estimation of relative sperm competition levels. Sperm velocity was also not correlated with midpiece size. The ecological and morphological functions of the midpiece were not determined in the present study.

We showed that sperm motility was strongly dependent on the fertilization mode. In the current study, the sperm of externally fertilizing species were motile only in seawater, except for that of *A. flavidus*, which sperm were motile in both seawater and isotonic solutions. This result suggests that marine fish with external fertilization have adapted to the external environment, that is, seawater, where eggs are fertilized. However, similar to the motility of *A. flavidus*, several externally fertilizing marine/freshwater sculpins exhibit sperm motility in both seawater/freshwater and isotonic solutions (Hayakawa & Munehara, 1998; Ito et al., 2021; Petersen et al., 2005). Thus, some species with external fertilization may be able to move in the ovarian fluid, generating the foothold of internal fertilization (Ito et al., 2021). By contrast, all sperm of internally fertilizing species, including the IGA species in the current study, progressed only in isotonic solution but not in seawater. Similar results have been reported for marine sculpins with IGA (Abe & Munehara, 2007; Koya et al., 1993). In addition, the sperm of freshwater fish with viviparity were only motile in isotonic solution but immotile in low osmotic solution in Baikal sculpin (Ito et al., 2021) and swordtail (Yang et al., 2006). Therefore, the sperm of the internal inseminator may have adapted to the internal environment. A previous study showed that the sperm of marine fish can swim in media over a comparatively larger range of osmolarity than that of freshwater fish (Alavi & Cosson, 2006). However, little information is available on sperm motility in marine fish subjected to internal fertilization. Based on a previous study on externally fertilizing marine fish (Alavi & Cosson, 2006) and our results, we conclude that the sperm motility of marine fish with internal fertilization and IGA is restricted to an isotonic solution and does not require motility in seawater because of the evolution of fertilization modes.

In the present study, even externally fertilizing *P. nagasakiensis* had a relatively sizeable genital papilla. A previous field study has shown that the genital papilla is used for ejaculation by protruding to rub eggs during mating (Moyer, 1975). With a few exceptions, such as *P. nagasakiensis*, externally fertilizing species do not require large genitalia. By contrast, the large genital organ was not associated with internal fertilization, contrary to popular belief that: two internal fertilizers (*D. temmincki temmincki* and *S. cheni*) did not have large genitalia for copulation, suggesting that copulatory organs may not be necessary for internal insemination in fish. These results suggest that even internally fertilizing fish may copulate in various ways, not simply using their genitalia, and have evolved a peculiar way of transferring sperm. The sea raven *Hemitripterus villosus*, which exhibits IGA, lacks large genitalia but ejaculates semen toward the genital duct projecting from a female for copulation (Munehara, 1996). Viviparous eelpouts (Zoarcidae) also do not have large genitalia and copulate through direct genital contact, although the details of their mating behavior are still unknown (Yao & Crim, 1995). More detailed observations of copulation behavior are necessary to clarify the variation in sperm transfer. Several hypotheses have been proposed regarding the evolution of large genitalia and their diversity, including male–male competition, female choice, and predation risk (Brennan & Prum, 2015; Heinen‐Kay & Langerhans, 2013; Langerhans, 2011). However, future work should also focus on animals that do not have genitalia despite performing internal fertilization in order to improve our understanding of the evolution of internal fertilization.

## 5 CONCLUSION

In this study, we compared the sperm morphology and motility of external fertilizers and species with internal insemination among close relatives to control for phylogenetic effects and in phylogenetic comparative methods. Although the sample size was limited and statistical power was not strong, we demonstrated that internal fertilization, including IGA, may influence sperm head morphology and motility in the extracellular environment. Total sperm length (including flagellum length) and velocity may be associated with sperm competition, but not fertilization mode, although there are a few exceptions. Previous studies have suggested that internal fertilizers have longer sperm than external fertilizers (Franzén, 1970; Lüpold & Pitnick, 2018; Stockley et al., 1996); however, our paired comparison and phylogenetic comparative analyses suggest that internal fertilization with internal insemination and IGA may have a little evolutionary effect on total sperm length when comparing closely related species and considering sperm competition levels.

## AUTHOR CONTRIBUTIONS

**Takeshi Ito:** Formal analysis (lead); funding acquisition (equal); investigation (equal); methodology (equal); visualization (lead); writing – original draft (lead). **Masaya Morita:** Formal analysis (supporting); investigation (supporting); writing – review and editing (equal). **Seiya Okuno:** Formal analysis (lead); methodology (supporting); writing – review and editing (supporting). **Kazuo Inaba:** Formal analysis (supporting); writing – review and editing (supporting). **Kogiku Shiba:** Formal analysis (supporting); writing – review and editing (supporting). **Hiroyuki Munehara:** Investigation (supporting); writing – review and editing (supporting). **Yasunori Koya:** Investigation (supporting); writing – review and editing (supporting). **Mitsuo Homma:** Investigation (supporting); writing – review and editing (supporting). **Satoshi Awata:** Formal analysis (lead); funding acquisition (lead); investigation (equal); supervision (lead); writing – review and editing (lead).

## CONFLICT OF INTEREST

The authors declare that they have no competing interests.

## Supporting information


Figure S1

Figure S2

Figure S3

Table S1

Table S2

Table S3

Table S4

Table S5

Table S6

## Data Availability

The datasets used and analyzed in the current study are shown in Tables S2 and S3.
