# Supplementary figures and images for: Fertilization modes and the evolution of sperm characteristics in marine fishes: Paired comparisons of externally and internally fertilizing species
Source: Ecol Evol. 2022 Dec 4;12(12):e9562. doi: 10.1002/ece3.9562 (PMC9720005; doi:10.1002/ece3.9562)

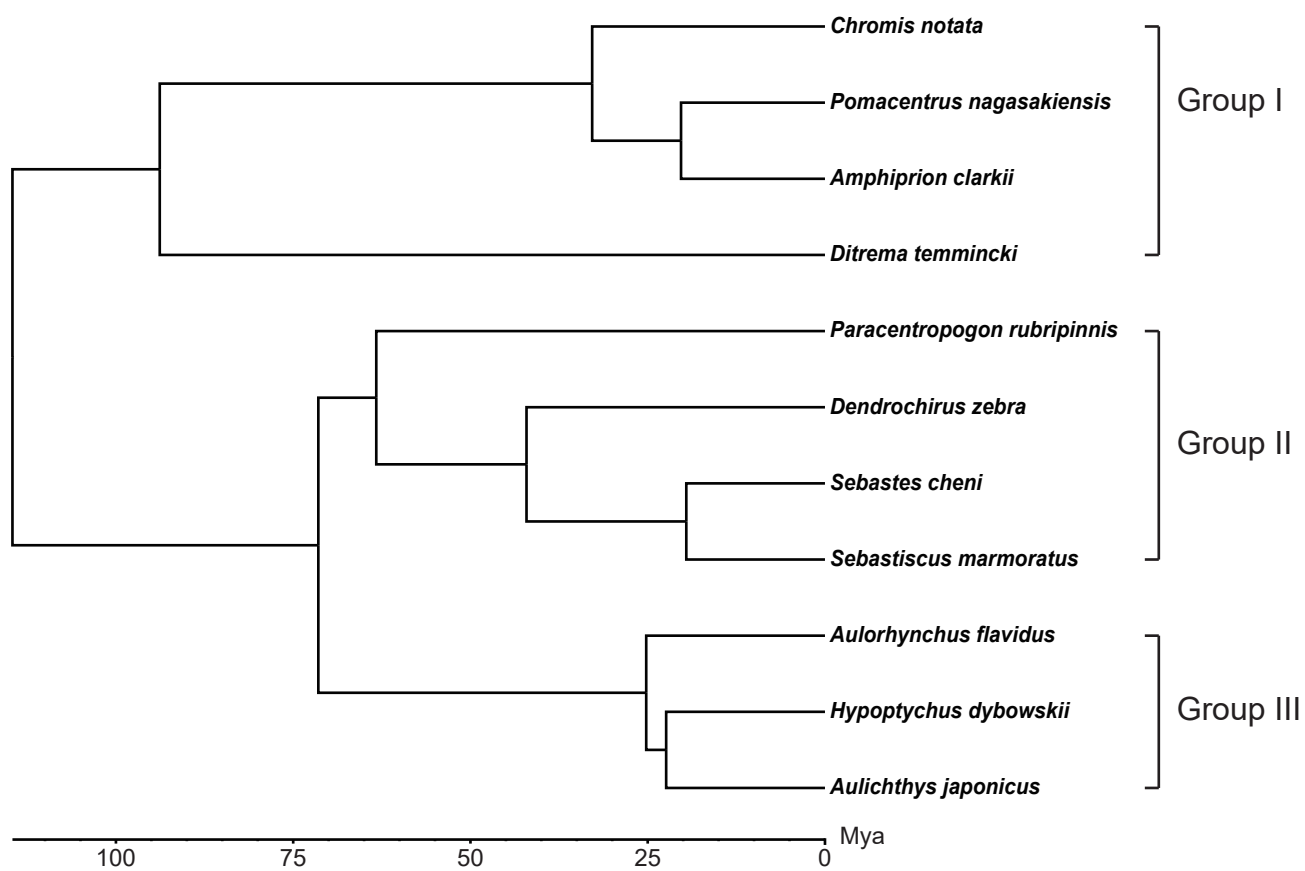

Supplement: Supplementary file 1 — Figure S1 [file ECE3-12-e9562-s003.pdf]

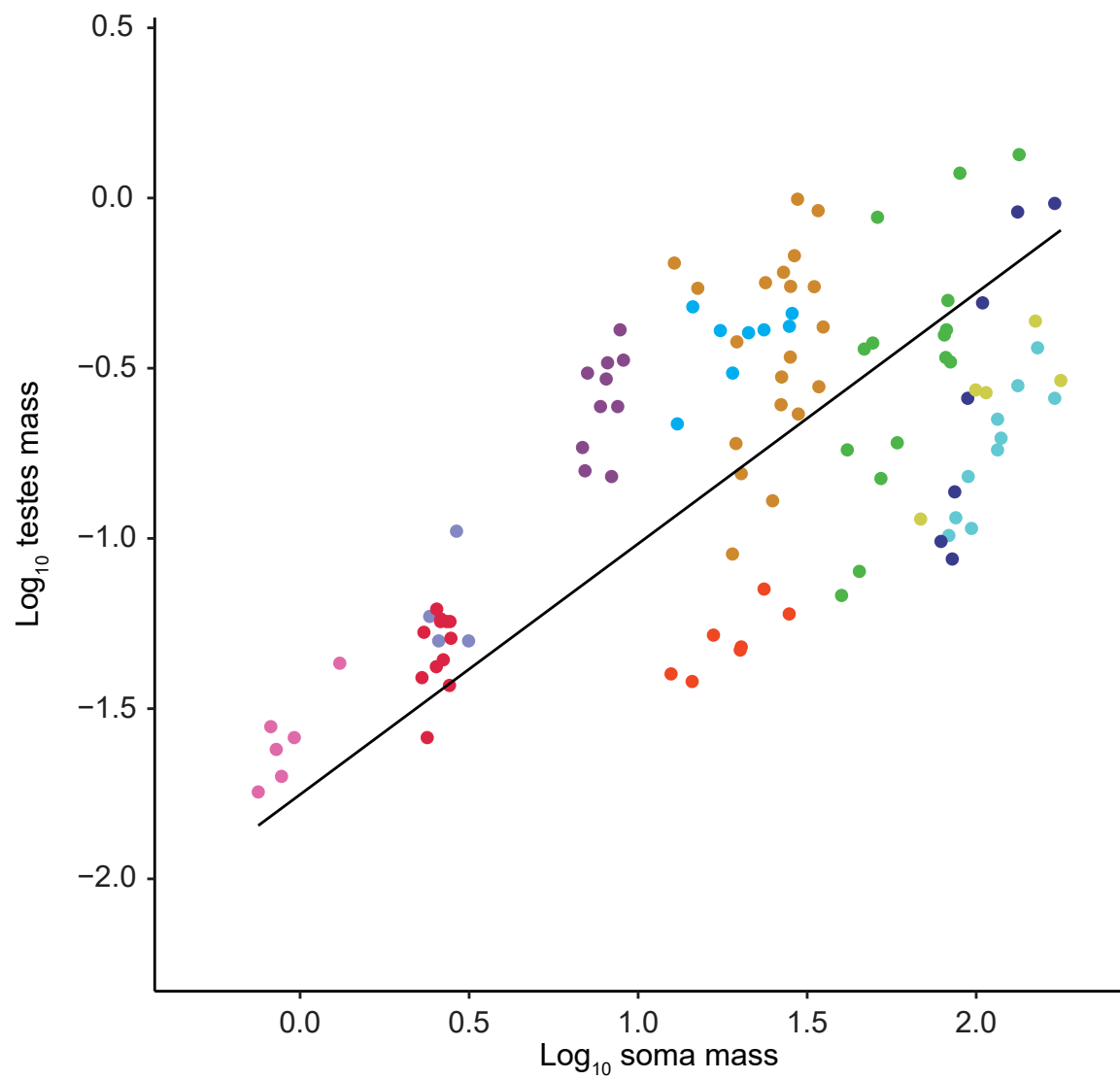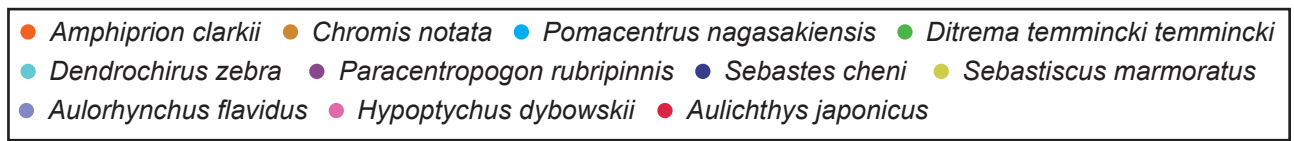

Supplement: Supplementary file 2 — Figure S2 [file ECE3-12-e9562-s001.pdf]
