# Supplementary material for: Fertilization modes and the evolution of sperm characteristics in marine fishes: Paired comparisons of externally and internally fertilizing species
Source: Ecol Evol. 2022 Dec 4;12(12):e9562. doi: 10.1002/ece3.9562 (PMC9720005; doi:10.1002/ece3.9562)
Supplement: Supplementary file 3 — Figure S3 [file ECE3-12-e9562-s007.pdf]

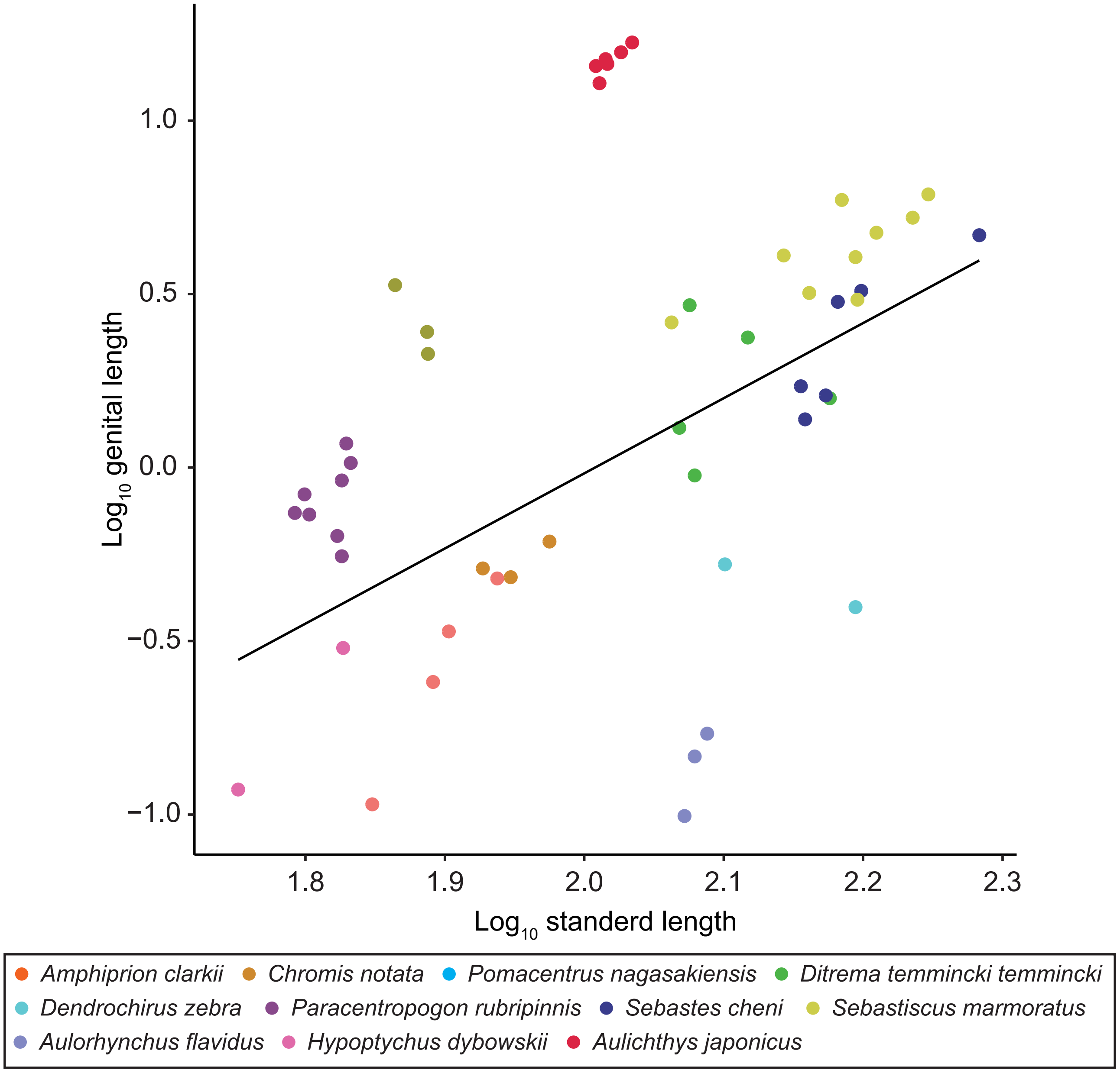

1.0
0.5
0.0
−0.5
−1.0
Log10 genital length
1.8
1.9
2.0
2.1
2.2
2.3
Log10 standerd length
Amphiprion clarkii
Chromis notata
Pomacentrus nagasakiensis
Ditrema temmincki temmincki
Dendrochirus zebra
Paracentropogon rubripinnis
Sebastes cheni
Sebastiscus marmoratus
Aulorhynchus flavidus
Hypoptychus dybowskii
Aulichthys japonicus
