# Supplementary material for: Fertilization modes and the evolution of sperm characteristics in marine fishes: Paired comparisons of externally and internally fertilizing species
Source: Ecol Evol. 2022 Dec 4;12(12):e9562. doi: 10.1002/ece3.9562 (PMC9720005; doi:10.1002/ece3.9562)
Supplement: Supplementary file 4 — Table S1 [file ECE3-12-e9562-s004.docx]

Table S1 Sampling information of fishes used for sperm analyses in this study.

| Group | Species | Male | Female | Sampling location^1^ | Depth (m) | WT (˚C) | Sampling date |
| --- | --- | --- | --- | --- | --- | --- | --- |
| I | *Amphiprion clarkii* | 7 | 0 | Ainan | 3-6 | 23.6-25.0 | Jul. 2018 |
|  | *Chromis notata* | 15 | 0 | Sado Island | 6-16 | 25.0-27.5 | Aug. 2015, Sep. 2016 |
|  | *Pomacentrus nagasakiensis* | 8 | 0 | Ainan | 3-6 | 23.2-25.4 | Jul. 2018, 2019 |
|  | *Ditrema temmincki temmincki* | 11 | 1 | Sado Island | 1-3 | 17.4-18.0 | Oct.-Nov. 2015, 2016, 2017 |
| II | *Dendrochirus zebra* | 8 | 0 | Ainan; Sesoko Island | 3-10 | 23.2-29.5 | Jul. 2016, 2019 |
|  | *Paracentropogon rubripinnis* | 9 | 0 | Sado Island | 12-15 | 20 | Jul. 2021 |
|  | *Sebastes cheni* | 7 | 1 | Sado Island | 1-5 | 9.7-11.2 | Jan.-Feb. 2019 |
|  | *Sebastiscus marmoratus* | 7 | 0 | Shimoda, Osaka bay | 5-10 | 10-11.2 | Jan. 2019 |
| III | *Aulorhynchus flavidus* | 4^2^ | 0 | Monterey, Tokyo Sea Life Park | 13-14 | 11.6 | Mar. 2018, Jul. 2021 |
|  | *Hypoptychus dybowskii* | 6 | 0 | Hakodate | 2-5 | 8.0 | Apr.2015, May 2017 |
|  | *Aulichthys japonicus* | 12 | 1 | Sado Island | 5-10 | 11.6-12.1 | Apr. 2016, Mar. 2017 |

^1^Ainan: Ainan, Ehime, Japan; Sado Island: Sado, Niigata, Japan; Sesoko Island: Sesoko, Okinawa, Japan; Shimoda: Shimoda, Shizuoka, Japan; Osaka Bay: Osaka, Japan; Monterey: Monterey, CA, USA; Hakodate: Hakodate, Hokkaido, Japan. ^2^Three of the four *Aulorhynchus flavidus* individuals were breeding individuals from Aquamarine Fukushima, Fukushima, Japan. WT: water temperature
