# Supplementary material for: Fertilization modes and the evolution of sperm characteristics in marine fishes: Paired comparisons of externally and internally fertilizing species
Source: Ecol Evol. 2022 Dec 4;12(12):e9562. doi: 10.1002/ece3.9562 (PMC9720005; doi:10.1002/ece3.9562)
Supplement: Supplementary file 7 — Table S4 [file ECE3-12-e9562-s005.docx]

| Sperm characteristics/outlier | *Amphiprion clarkii* | *Chromis notata* | *Pomacentrus nagasakiensis* | *Ditrema temmincki temmincki* | Statistics | |
| --- | --- | --- | --- | --- | --- | --- |
|  |  |  |  |  | χ^2^_3_ | *P* |
| Outlier of sperm morphology (n) | 7 | 6 | 3 | 6 |  |  |
| Total sperm length (μm) | 32.67 ± 2.29^a^ (7, 117) | 30.91 ± 1.29^b^ (9, 173) | 25.91 ± 2.01^c^ (8, 108) | 43.71 ± 0.87^d^ (9, 123) | 98.16 | < 0.0001 |
| Flagella length (μm) | 30.08 ± 2.28^a^ (7, 114) | 29.43 ± 1.25^a^ (9, 126) | 23.72 ± 1.85^b^ (8, 71) | 40.12 ± 0.88^c^ (9, 119) | 77.73 | < 0.0001 |
| Head length (μm) | 2.65 ± 0.05^a^ (7, 117) | 1.65 ± 0.10^b^ (9, 128) | 2.15 ± 0.16^c^ (8, 73) | 3.59 ± 0.08^d^ (9, 124) | 136.27 | < 0.0001 |
| Head width (μm) | 2.67 ± 0.07^a^ (7, 114) | 2.50 ± 0.10^b^ (9, 126) | 2.28 ± 0.24^c^ (8, 75) | 1.28 ± 0.07^d^ (9, 126) | 100.43 | < 0.0001 |
| Midpiece length (μm) | 0.89 ± 0.12^a^ (7, 96) | 0.99 ± 0.07^a^ (9, 130) | 0.90 ± 0.18^a^ (8, 61) | 3.40 ± 0.27^b^ (9, 121) | 125.23 | < 0.0001 |
| Midpiece width (μm) | 2.60 ± 0.26^a^ (7, 96) | 1.05 ± 0.10^b^ (9, 130) | 1.28 ± 0.18^c^ (8, 61) | 1.14 ± 0.10^bc^ (9, 124) | 93.93 | < 0.0001 |
| Head length / head width | 0.99 ± 0.03^a^ (7, 111) | 0.66 ± 0.02^b^ (9, 121) | 1.02 ± 0.14^c^ (8, 73) | 2.83 ± 0.15^d^ (9, 122) | 146.62 | < 0.0001 |
| Midpiece length / midpiece width | 0.36 ± 0.05^a^ (7, 92) | 0.96 ± 0.07^b^ (9, 126) | 0.71 ± 0.10^c^ (8, 58) | 3.06 ± 0.42^d^ (9, 121) | 105.02 | < 0.0001 |
| Outlier of sperm velocity (n) | 5 | 3 | 1 | 4 |  |  |
| Sperm velocity (μm/s) | 71.26 ± 11.60^a^ (7, 184) | 106.74 ± 29.57^bc^ (7, 153) | 112.14 ± 21.22^b^ (7, 172) | 89.28 ± 11.63^c^ (10, 145)  Sperm from ovary:  86.8±19.97 (1, 23) | 17.68 | 0.0005 |

**Table S4** Summary of sperm characteristics in group I.

All values represent the mean ± SD. Numbers of individuals (left) and sperm (right) used for the analyses are shown in parentheses. Different superscripts indicate significant differences in each sperm characteristic between species (LMMs with sequential Bonferroni correction, *P* < 0.05).
