# Supplementary material for: Fertilization modes and the evolution of sperm characteristics in marine fishes: Paired comparisons of externally and internally fertilizing species
Source: Ecol Evol. 2022 Dec 4;12(12):e9562. doi: 10.1002/ece3.9562 (PMC9720005; doi:10.1002/ece3.9562)
Supplement: Supplementary file 9 — Table S6 [file ECE3-12-e9562-s009.docx]

| Sperm characteristics/outlier | *Aulorhynchus flavidus* | *Hypoptychus dybowskii* | *Aulichithys japonicus* | Statistics | |
| --- | --- | --- | --- | --- | --- |
|  |  |  |  | χ^2^_2_ | *P* |
| Outlier of sperm morphology (n) | 3 | 4 | 8 |  |  |
| Total sperm length (μm) | 35.38 ± 1.60^a^ (4, 64) | 39.98 ± 0.48^b^ (4, 58) | 37.66 ± 0.77^c^ (7, 174) | 22.47 | < 0.0001 |
| Flagella length (μm) | 32.75 ± 1.73^a^ (4, 62) | 37.77 ± 0.54^b^ (4, 55) | 35.28 ± 0.67^c^ (7, 168) | 23.89 | < 0.0001 |
| Head length (μm) | 2.60 ± 0.16^a^ (4, 65) | 2.24 ± 0.15^b^ (4, 58) | 2.39 ± 0.16^b^ (7, 176) | 9.91 | 0.007 |
| Head width (μm) | 1.71 ± 0.27^a^ (4, 66) | 1.16 ± 0.08^b^ (4, 59) | 1.17 ± 0.05^b^ (7, 180) | 22.02 | < 0.0001 |
| Midpiece length (μm) | 0.88 ± 0.13^a^ (4, 65) | 0.61 ± 0.07^a^ (4, 60) | 1.59 ± 0.35^b^ (7, 176) | 24.89 | < 0.0001 |
| Midpiece width (μm) | 1.10 ± 0.12^ab^ (4, 66) | 1.01 ± 0.08^a^ (4, 59) | 1.17 ± 0.11^b^ (7, 171) | 7.93 | 0.019 |
| Head length / head width | 1.57 ± 0.27^a^ (4, 64) | 1.96 ± 0.15^b^ (4, 55) | 2.06 ± 0.14^b^ (7, 174) | 14.30 | 0.0008 |
| Midpiece length / midpiece width | 0.82 ± 0.03^a^ (4, 64) | 0.61 ± 0.08^a^ (4, 57) | 1.38 ± 0.30^b^ (7, 165) | 22.32 | < 0.0001 |
| Head cross-sectional area (μm^2^) | 1.23±0.21^a^ (4, 64) | 1.54±0.12^b^ (4, 55) | 1.61±0.11^b^ (7, 174) | 14.30 | 0.0007 |
| Outlier of sperm velocity (n) | 2 | 2 | 2 |  |  |
| Sperm velocity (μm/s) | 85.79 ± 13.51^a^ (4, 78) | 49.08 ± 22.85^b^ (6, 71) | 55.60 ± 6.21^b^ (7, 104)  Sperm from ovary:  51.14±3.58 (1, 3) | 11.70 | 0.003 |

**Table S6** Summary of sperm characteristics in group III.

All values represent the mean ± SD. Numbers of individuals (left) and sperm (right) used for the analyses are shown in parentheses. Different superscripts indicate significant differences in each sperm characteristic between species (LMMs with sequential Bonferroni correction, *P* < 0.05). The head cross-sectional areas were calculated according to Ito et al. (2021).
